# Evidence for bivariate linkage of obesity and HDL-C levels in the Framingham Heart Study

**DOI:** 10.1186/1471-2156-4-S1-S52

**Published:** 2003-12-31

**Authors:** Rector Arya, Donna Lehman, Kelly J Hunt, Jennifer Schneider, Laura Almasy, John Blangero, Michael P Stern, Ravindranath Duggirala

**Affiliations:** 1 Division of Clinical Epidemiology, Department of Medicine, University of Texas Health Science Center, San Antonio, Texas, USA; 2 Department of Genetics, Southwest Foundation for Biomedical Research, San Antonio, Texas, USA

## Abstract

**Background:**

Epidemiological studies have indicated that obesity and low high-density lipoprotein (HDL) levels are strong cardiovascular risk factors, and that these traits are inversely correlated. Despite the belief that these traits are correlated in part due to pleiotropy, knowledge on specific genes commonly affecting obesity and dyslipidemia is very limited. To address this issue, we first conducted univariate multipoint linkage analysis for body mass index (BMI) and HDL-C to identify loci influencing variation in these phenotypes using Framingham Heart Study data relating to 1702 subjects distributed across 330 pedigrees. Subsequently, we performed bivariate multipoint linkage analysis to detect common loci influencing covariation between these two traits.

**Results:**

We scanned the genome and identified a major locus near marker D6S1009 influencing variation in BMI (LOD = 3.9) using the program SOLAR. We also identified a major locus for HDL-C near marker D2S1334 on chromosome 2 (LOD = 3.5) and another region near marker D6S1009 on chromosome 6 with suggestive evidence for linkage (LOD = 2.7). Since these two phenotypes have been independently mapped to the same region on chromosome 6q, we used the bivariate multipoint linkage approach using SOLAR. The bivariate linkage analysis of BMI and HDL-C implicated the genetic region near marker D6S1009 as harboring a major gene commonly influencing these phenotypes (bivariate LOD = 6.2; LOD_eq _= 5.5) and appears to improve power to map the correlated traits to a region, precisely.

**Conclusions:**

We found substantial evidence for a quantitative trait locus with pleiotropic effects, which appears to influence both BMI and HDL-C phenotypes in the Framingham data.

## Background

The incidence rates of complex diseases such as obesity and type 2 diabetes have been increasing worldwide [1,2]. Obesity is a major risk factor for type 2 diabetes, hypertension, dyslipidemia, and other cardiovascular complications and it has become a global public health problem [3]. Several epidemiological studies have shown that obesity and low levels of high-density lipoprotein cholesterol (HDL-C) are major risk factors for coronary heart disease [4,5] and there is substantial evidence that obesity and HDL-C are strongly influenced by genetic factors. In fact, a number of studies have identified chromosomal regions harboring quantitative trait loci (QTL) influencing obesity [6] or dyslipidemia [7]. Surprisingly, little is known about specific loci commonly influencing obesity and HDL-C concentrations, despite the evidence for appreciable correlation between these phenotypes. The present analysis examines whether there exists any chromosomal regions harboring genes that influence the covariation between BMI and HDL-C phenotypes using the Framingham Heart Study data through a bivariate linkage approach.

## Subjects and Methods

### The Framingham Heart Study

For purposes of the current analysis, Framingham Heart Study participants from the original cohort were combined with participants from the offspring cohort to maximize the number of individuals per pedigree. Information collected as part of the 12^th ^examination (except for height, which was collected at the 14^th ^examination) of the original cohort, which occurred 1970 to 1971, was combined with information collected during the first examination of the offspring cohort, which occurred from 1971 to 1975. Of the more than 10,000 participants enrolled in either the Framingham Heart Study or the Framingham Offspring Study, genotypic information was available on 1702 individuals from 330 extended pedigrees. The pedigrees include from 2 to 29 genotyped individuals and the genotyped sample includes 394 individuals from the original cohort and 1308 individuals from the offspring cohort.

### Phenotypes

We used BMI and HDL-C data collected as part of the Framingham Heart Study. BMI was calculated as weight (in kilograms)/height squared (in meters). HDL-C (mg/dl) was measured by automated enzymatic methods. BMI and HDL-C values were log transformed to minimize the problem of non-normality.

### Variance components linkage analysis

A multipoint variance components linkage analysis was used to test linkage between marker loci and a given phenotype, which was based on specifying the expected genetic covariances between pairs of relatives as a function of their identity by descent (IBD) at a marker linked to a QTL [8]. It allows for locus-specific effects, residual genetic effects, covariate effects, and random environmental effects. Since the trait-specific linkage analysis (i.e., univariate) cannot exploit the additional information embedded in the correlation pattern between two quantitative traits, a bivariate multipoint linkage analysis was used to exploit the additional information contained in the correlation pattern between two quantitative traits [9,10].

### Univariate genetic linkage analysis

In a simple additive model in which *n *QTLs and an unknown number of residual polygenes influence a given trait, the covariance matrix (Ω) for a pedigree is given by



where Π_i _is a matrix whose elements (π_ijl_) provide the expected proportion of genes that individuals *j *and *l *share IBD at a QTL (q_i_) that is linked to a genetic marker locus, σ^2^_q _is the additive genetic variance due to the major locus, Φ is the kinship matrix, σ^2^_g _is the genetic variance due to additive genetic effects, I is an identity matrix, and σ^2^_e _is the variance due to random environmental effects.

### Bivariate genetic linkage analysis

The extension of the variance components linkage approach to the bivariate situation facilitates the testing of linkage of two correlated phenotypes to a single genetic region simultaneously. In the bivariate analysis, trait-specific means, variance components relating to major gene effects (σ^2^_q_), residual additive genetic effects (σ^2^_g_), random environmental effects (σ^2^_e_), covariate effects (age and sex terms), as well as the three associated correlations ρ_q _(correlation caused by a major gene), ρ_g _(correlation caused by residual additive genetic effects), and ρ_e _(correlation caused by random environmental effects) are estimated simultaneously using maximum-likelihood estimates. Using this bivariate model, we tested the null hypothesis that σ^2^_q _= 0 for both traits by comparing the log likelihood of this restricted model to that of a model in which σ^2^_q _was estimated for the traits. In addition, tests for the hypotheses of complete pleiotropy (i.e., the same major gene in the chromosomal region of interest affects both traits) and coincident linkage (i.e., no shared major gene effects in the chromosomal region of interest on the two traits) can be performed [9]. To test complete pleiotropy or coincident linkage, likelihood for the linkage model in which ρ_q _was estimated was compared with the model in which ρ_q _was constrained to -1 (complete pleiotropy [a negative value for ρ_q _was chosen since the overall polygenic correlation between the examined traits was found to be negative]) or to 0 (coincident linkage [i.e., ρ_q _= 0]), respectively. These analytical techniques were implemented in the computer program SOLAR [8].

## Results

Descriptive statistics for the examined phenotypes are reported in Table 1. To minimize the problem of non-normality, BMI and HDL-C values were log transformed. Both phenotypes exhibited high heritabilities (h^2 ^± SE, *ln *BMI = 0.45 ± 0.05 and *ln *HDL-C = 0.52 ± 0.06), after adjusting for age and sex influences.

**Figure 1:**
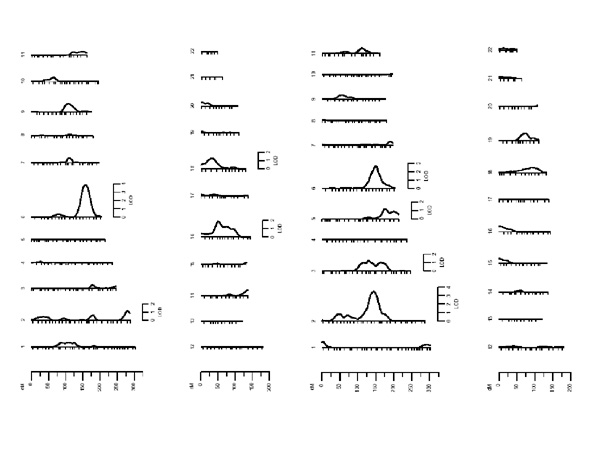
Chromosomal regions linked to *ln *BMI (A) and *ln *HDL-C (B) in genomic scans

As shown in Figure 1, univariate multipoint linkage analysis yielded significant evidence for linkage with *ln *BMI (LOD = 3.9) and suggestive evidence with *ln *HDL-C (LOD = 2.7, 150 cM) on chromosome 6. The chromosomal region at 158 cM near markers D6S1009 and GATA184A08 on chromosome 6 showed evidence for linkage to both BMI and HDL-C, although the trait-specific linkage curves peaked at two adjacent locations covering about an 8cM region (Figure 1). Both genetic (-0.26) and environmental (-0.17) correlations between BMI and HDL-C were low. Subsequently, we extended our analytical approach to the bivariate situation to exploit the additional information underlying the pattern of covariation between *ln *BMI and *ln *HDL-C phenotypes.

**Figure 2:**
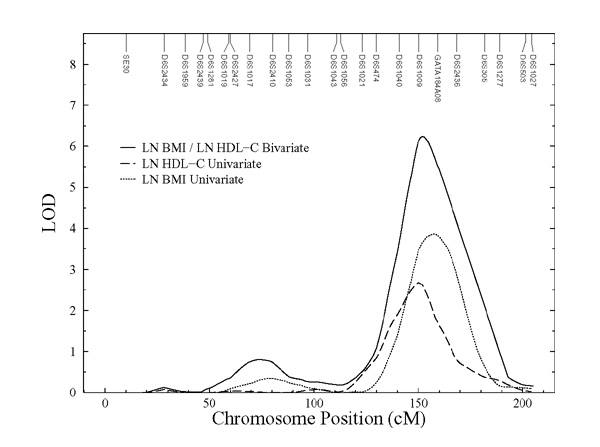
Univariate and bivariate linkage profiles for *ln *BMI and *ln *HDL-C phenotypes

Table 2 presents the results from multipoint bivariate linkage analyses of *ln *BMI and *ln *HDL-C. A bivariate LOD score of 6.24 was obtained for the region involving the ordered markers D6S1009-GATA184A08 for the trait pair of *ln *BMI-*ln *HDL-C (Figure 2). The corresponding *p-*value is 1.8 × 10^-7 ^(λ = 28.75). The bivariate LOD score can be converted to univariate LOD equivalent (LOD_eq_) of 5.46 for this chromosomal region, which adds further significance to our finding. The correlation (ρ_q _± SE) between trait pair *ln *BMI-*ln *HDL-C due to QTL effects is -0.54 ± 0.19, which is statistically significant (*p *= 0.0072). It should be noted that the test for complete pleiotropy (i.e., locus-specific correlation between *ln *BMI and *ln *HDL is not significantly different from -1) was rejected (*p *= 0.0166). However, coincident linkage (i.e., locus-specific correlation between *ln *BMI and *ln *HDL is equal to 0) for *ln *BMI and *ln *HDL was strongly rejected (*p *= 0.0072).

## Discussion

The univariate linkage analyses implicated ~8cM chromosomal region, involving the sequential markers D6S1009-GATA184A08, on chromosome 6q influencing both *ln *BMI and *ln *HDL-C. The genetic correlation between *ln *BMI and *ln *HDL was higher compared with environmental correlation and showed an inverse relationship as expected. This pattern of correlation is consistent with previous observations in which low overall genetic correlations were observed between obesity and lipid measures in a Mexican-American population [11]. Since both *ln *BMI and *ln *HDL-C have exhibited significant linkage to the same region on chromosome 6q, we specifically examined the mapping pattern of the bivariate trait. The bivariate analysis has resulted in co-localization of these correlated traits at a chromosomal region near marker D6S1009. The tests for complete pleiotropy and coincident linkage indicated that the putative major gene influences both BMI and HDL-C, albeit incompletely. As remarked by Almasy et al. [9], such a situation of incomplete pleiotropy would be expected if either epistatic or gene × environmental interactions influence the putative major gene's effect on one of the two correlated traits, but not both. Also, it is possible that multiple variants in a putative major gene may differentially influence the correlated phenotypes.

It appears that the trait pairs with low to moderate overall genetic correlations (BMI vs. HDL-C) appear to contain much more information to improve power to co-localize precisely the correlated phenotypes to a genetic region. It is important to note that this same region (or genetic locations close to this region) on chromosome 6q has been reported to affect various components of the Insulin Resistance Syndrome including obesity, insulin, lipid, and blood pressure measures [e.g., [12-14]]. Recently, Atwood et al. [15] reported evidence for linkage of the same region on chromosome 6q23-25 (D6S1009-GATA184A08-D6S2436-D6S305) with six separate measurements of BMI with corresponding maximum LOD scores of 4.64, 2.29, 2.41, 1.40, 0.99, and 3.08, respectively, collected across 28 years of the Framingham Heart Study. This study provides additional evidence in support of the implicated region on chromosome 6 harboring the QTL affecting BMI variation in Framingham data.

## Conclusions

Using a bivariate linkage approach, we have found strong evidence for a QTL, which appears to influence both BMI and HDL-C, in Framingham data. Our study suggests that the putative locus lies in the chromosomal region near marker D6S1009 on chromosome 6.

## Figures and Tables

**Table 1:** Descriptive statistics

**Variable**	***N***	**Mean**	**SD**	**Kurtosis**	**Skewness**
Age	1702	38.4	14.6	-	-
BMI	1686	25.3	4.2	2.5	0.9
HDL-C	1294	50.7	14.2	0.9	0.7
*ln *BMI	1686	3.2	0.2	0.6	0.3
*ln *HDL-C	1294	3.9	0.3	0.2	-0.2

**Table 2:** Results from bivariate linkage analyses of the trait pair *ln *BMI-*ln *HDL-C phenotypes

**Trait Pair**	**cM^A^**	**ρ_q_^B^**	**Bivariate LOD**	**λ^C^**	***P* ^D^**	**LOD_eq_^E^**
*ln *BMI-*ln *HDL	152	-0.54	6.24	28.75	1.8 × 10^-7^	5.46

^A^Distance from pter; ^B^ρ_q _is correlation due to QTL effects; ^C^likelihood-ratio statistic λ. ^D^Asymptotic *P *value, under the assumption that λ is distributed as a 1/4X^2^_2 _: 1/2X^2^_1 _: 1/4X^2^_0 _mixture. ^E^Lod_eq _is the univariate lod score corresponding to the reported bivariate lod score
